# Supplementary material for: Host receptor-targeted therapeutic approach to counter pathogenic New World mammarenavirus infections
Source: Nat Commun. 2022 Jan 28;13:558. doi: 10.1038/s41467-021-27949-3 (PMC8799657; doi:10.1038/s41467-021-27949-3)
Supplement: Supplementary file 1 — Supplementary Information File [file 41467_2021_27949_MOESM1_ESM.pdf]

Supplementary Information

Host Receptor-Targeted Therapeutic Approach to Counter Pathogenic New World Mammarenavirus Infections

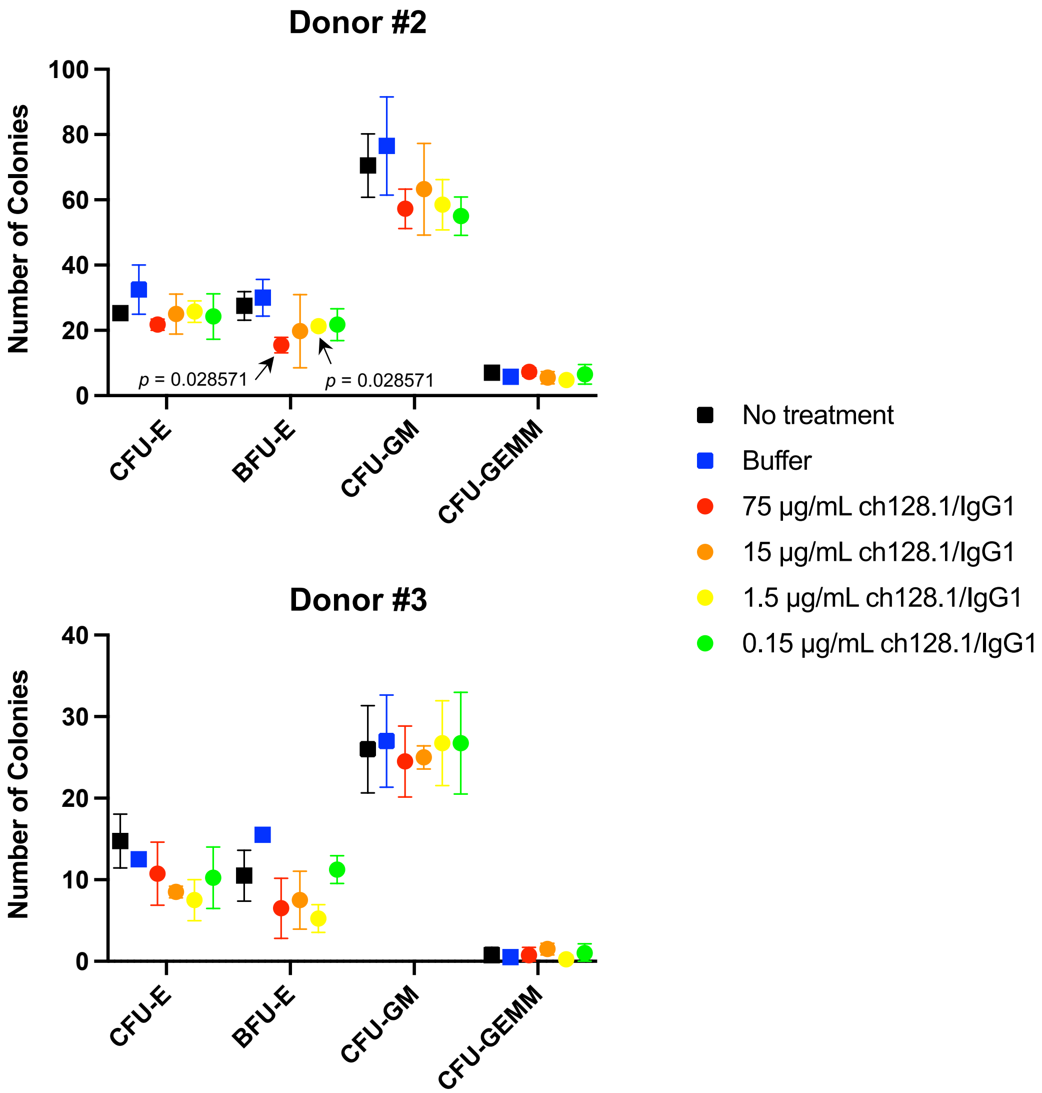

Supplementary Figure 1. Evaluation of the potential toxicity of ch128.1/IgG1 to committed hematopoietic progenitor cells. Human bone marrow mononuclear cells (BMMC) were treated with ch128.1/IgG1 at various concentrations and cultured for 14 days. Colony number data represent the mean ± standard error of quadruplicate samples, except for the buffer and 15 μg/mL samples for donor #3 for which the date represent the mean ± standard error of duplicate samples. The data shown are from 2 independent experiments using BMMC from 2 different donors. Results from donor #1 are shown in Figure 6. Statistical significance compared to buffer alone treatment were determined using the non-parametric, unpaired Mann-Whitney test. No significant differences were observed for donor #3.

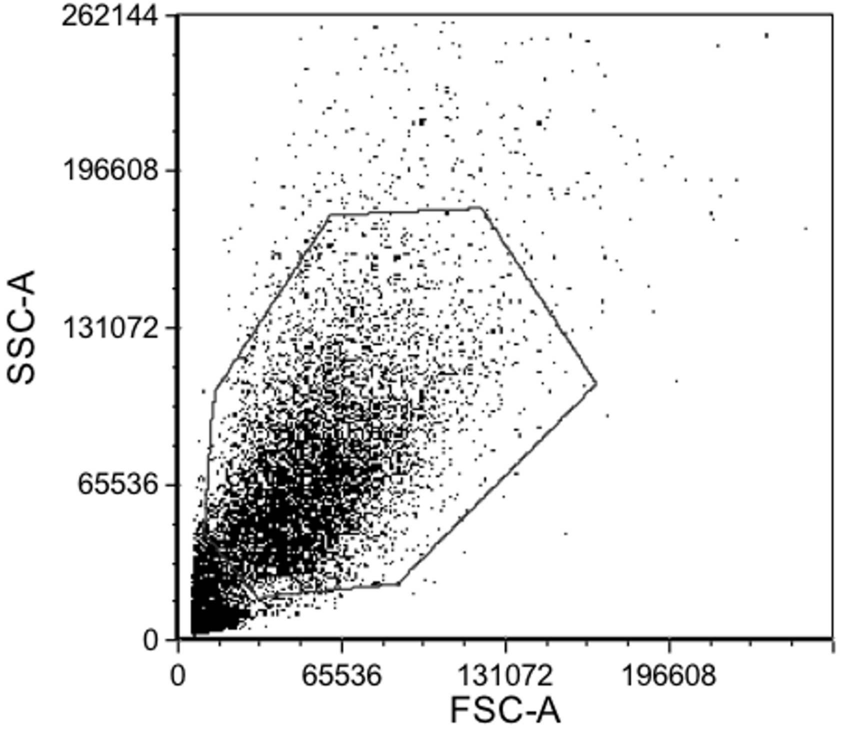

Supplementary Figure 2. Gating strategy for standard flow cytometry assays. Cell populations were identified and gated using forward scatter (FSC) and side scatter (SSC) density plots. This population gate (P1) was used to identify live cells and exclude debris (events with low forward and low side scatter). Analysis was performed in FSC Express version 3.0 (De Novo Software). This strategy corresponds to Figure 7, Figure 8 panels **a** and **b**, and Figure 9 panels **a** and **b**.

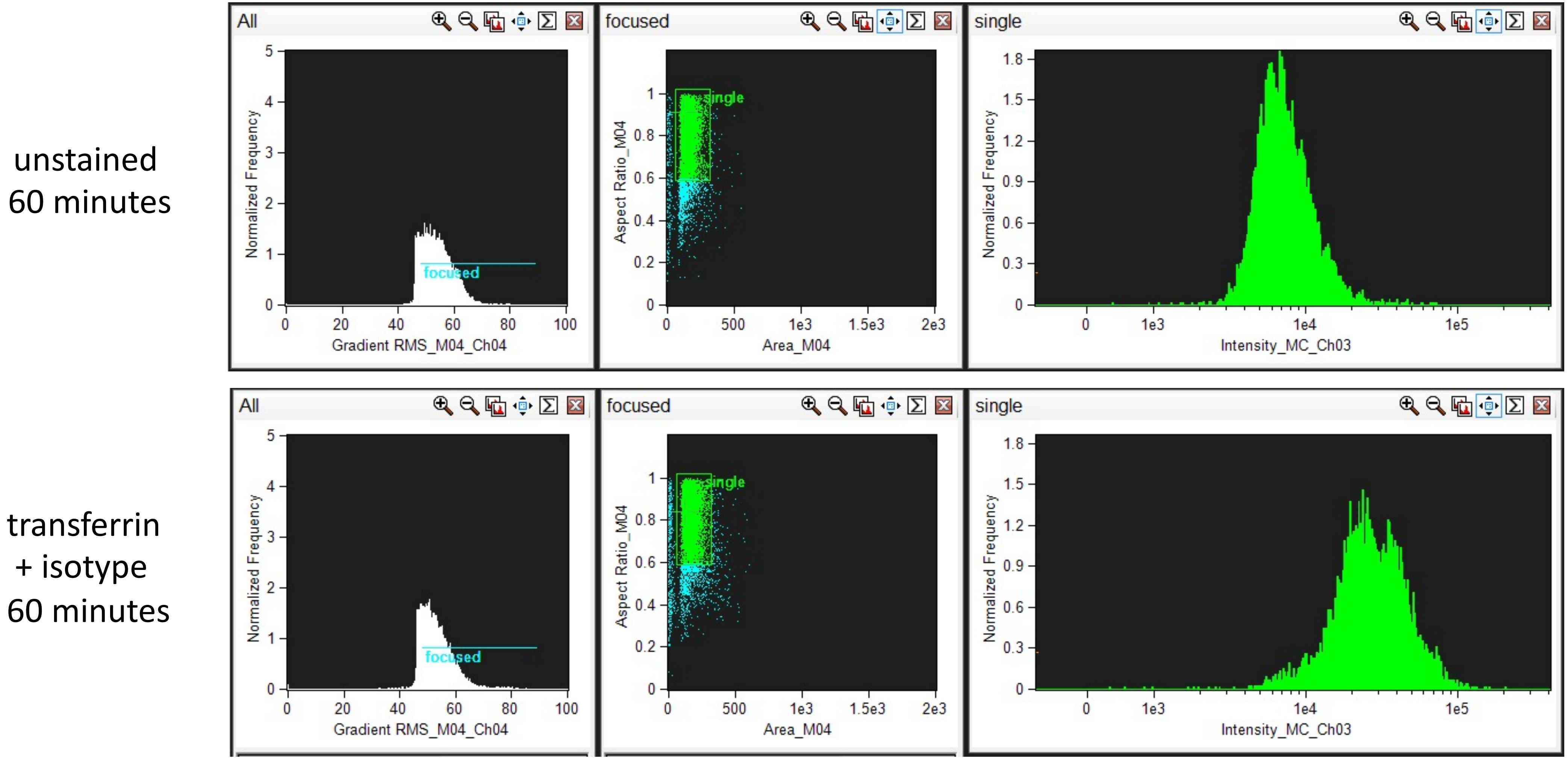

Supplementary Figure 3. Gating strategy for imaging flow cytometry assays. In focus events were gated by selecting events with a Gradient RMS value of greater than 50 on the brightfield channel. Single cell events were selected on the brightfield area versus aspect ratio density graphs, with single cells having an intermediate area value and a high aspect ratio value. Analysis was performed in the IDEAS software version 6.2 (Luminex Corporation). This strategy corresponds to Figures 8 panels **c-e** and Figure 9 panels **c-e**.
