# Supplementary material for: Host receptor-targeted therapeutic approach to counter pathogenic New World mammarenavirus infections
Source: Nat Commun. 2022 Jan 28;13:558. doi: 10.1038/s41467-021-27949-3 (PMC8799657; doi:10.1038/s41467-021-27949-3)
Supplement: Supplementary file 3 — Reporting Summary [file 41467_2021_27949_MOESM3_ESM.pdf]

nature research

Corresponding author(s): Brian B. Gowen, Manuel L. Penichet, Gustavo Helguera

Last updated by author(s): December 7, 2021

# Reporting Summary

Nature Research wishes to improve the reproducibility of the work that we publish. This form provides structure for consistency and transparency in reporting. For further information on Nature Research policies, see our Editorial Policies and the Editorial Policy Checklist.

## Statistics

For all statistical analyses, confirm that the following items are present in the figure legend, table legend, main text, or Methods section.

| n/a | Confirmed | |
|---|---|---|
| ☐ | ☒ | The exact sample size ($n$) for each experimental group/condition, given as a discrete number and unit of measurement |
| ☐ | ☒ | A statement on whether measurements were taken from distinct samples or whether the same sample was measured repeatedly |
| ☐ | ☒ | The statistical test(s) used AND whether they are one- or two-sided<br>*Only common tests should be described solely by name; describe more complex techniques in the Methods section.* |
| ☐ | ☒ | A description of all covariates tested |
| ☐ | ☒ | A description of any assumptions or corrections, such as tests of normality and adjustment for multiple comparisons |
| ☐ | ☒ | A full description of the statistical parameters including central tendency (e.g. means) or other basic estimates (e.g. regression coefficient) AND variation (e.g. standard deviation) or associated estimates of uncertainty (e.g. confidence intervals) |
| ☐ | ☒ | For null hypothesis testing, the test statistic (e.g. $F$, $t$, $r$) with confidence intervals, effect sizes, degrees of freedom and $P$ value noted<br>*Give P values as exact values whenever suitable.* |
| ☒ | ☐ | For Bayesian analysis, information on the choice of priors and Markov chain Monte Carlo settings |
| ☒ | ☐ | For hierarchical and complex designs, identification of the appropriate level for tests and full reporting of outcomes |
| ☒ | ☐ | Estimates of effect sizes (e.g. Cohen's $d$, Pearson's $r$), indicating how they were calculated |

*Our web collection on statistics for biologists contains articles on many of the points above.*

## Software and code

Policy information about availability of computer code

| | |
|---|---|
| Data collection | X-ray diffraction data were collected using beamline 24-ID-E (Advanced Photon Source, Argonne National Laboratory). For standard flow cytometry, FACSDiva™ acquisition software (version 8.0.3; BD Biosciences) was used. For imaging flow cytometry, the INSPIRE acquisition software (version 201.1.0.744; Luminex Corporation) was used. |
| Data analysis | Statistical analyses were performed GraphPad Prism software (version 9; GraphPad Software). The structure of Fab128.1/IgG1 was determined by molecular replacement using the Phaser suite of programs. Crystallographic refinement was performed using Phenix (version 1.8.4) and Buster (version 2.11.2), while structure visualization and modeling were performed in COOT (version 0.9.1) and PyMOL (version 2.3.4) (Schrödinger). For the docking analysis, the initial coordination of the Fab128.1/IgG1 to hTfR1 was performed using the ClusPro (version 2.0), and the docking model was obtained using the PyRosetta software suite (PyRosetta 3.5 release-84). Standard flow cytometry histograms were created using FCS Express (version 3.0; De Novo Software). For imaging flow cytometry, the IDEAS analysis software (version 6.2; Luminex Corporation) was used. |

For manuscripts utilizing custom algorithms or software that are central to the research but not yet described in published literature, software must be made available to editors and reviewers. We strongly encourage code deposition in a community repository (e.g. GitHub). See the Nature Research guidelines for submitting code & software for further information.

## Data

Policy information about availability of data

All manuscripts must include a data availability statement. This statement should provide the following information, where applicable:

- Accession codes, unique identifiers, or web links for publicly available datasets
- A list of figures that have associated raw data
- A description of any restrictions on data availability

Data Availability
The authors declare that the main data supporting the findings of this study are available within the article and its Supplementary Information files. Source data for Figures 1, 2, 4, 5 and 6 are provided with the paper. Additional data that support the findings of this study have been deposited in the Worldwide Protein Data Bank (wwPDB) with accession code: PDB ID 6WLA (https://doi.org/10.2210/pdb6WLA/pdb) and cited in the reference list (72). All other relevant data are available from the corresponding authors.

# Field-specific reporting

Please select the one below that is the best fit for your research. If you are not sure, read the appropriate sections before making your selection.

[x] Life sciences  [ ] Behavioural & social sciences  [ ] Ecological, evolutionary & environmental sciences

For a reference copy of the document with all sections, see nature.com/documents/nr-reporting-summary-flat.pdf

# Life sciences study design

All studies must disclose on these points even when the disclosure is negative.

| | |
|---|---|
| Sample size | For in vitro inhibition studies (Fig. 1), sample sizes were determined based on accepted standards of 3-4 biological replicates per experimental group. For the biolayer interferometry experiments (Fig. 2), no sample size calculations were performed to power the experiment, and no statistical methods were used to predetermine sample size. For the in vivo efficacy studies (Figs. 4 and 5), the number of mice per group was based on power analysis performed using commonly accepted values for type I error (0.05) and power (80%). For the progenitor assays (Fig. 6 and Supplementary Fig. 1), no sample size calculations were performed to power the experiment, and no statistical methods were used to predetermine sample size. The experiment was conducted according to the manufacturer's recommendations. The assay is normally performed with duplicates or triplicates so our use of quadruplicates is sufficient. This experiment was performed three times using BMMC from different donors. For the flow cytometry experiment (Fig. 7), no sample size calculations were performed to power the experiment, and no statistical methods were used to predetermine sample size. Flow cytometry assays are not performed in replicates due to the high number of acquisition events collected per sample (we collected data for 10,000 events, which is common for simple binding studies that were performed). For the imaging flow cytometry experiments (Figs. 8 and 9), no sample size calculations were performed to power the experiment, and no statistical methods were used to predetermine sample size. Flow cytometry assays (including imaging studies) are not performed in replicates due to the high number of acquisition events collected per sample (we collected data for 10,000 events, which is common for this type of imaging study). Multiple images are obtained for each event (brightfield and fluorescent images) so the amount of data collected is very large. |
| Data exclusions | No data were excluded from any of the analyses |
| Replication | Inhibitory activity of the ch128.1 antibody was reproduced versus 2 strains of Junin virus (Fig. 1). For the biolayer interferometry assessment, the experiment was performed twice with similar results (Fig. 2). Efficacy of the ch128.1 and mutant antibodies was reproduced in a second mouse efficacy experiment (Figs. 4 and 5). The progenitor assay was performed 3 times with 3 different BMMC donors with reproducible results (Fig. 6 and Supplementary Fig. 1). The standard flow cytometric study with MM.1S cells (Fig. 7) was performed twice with reproducible outcomes. Imaging flow cytometry experiments (Figs. 8 and 9) were performed one time at the 37 degree C incubation temperature. Repeat trials were performed at room temperature with similar results. Because the imaging flow cytometry assay was performed to confirm the standard flow cytometry data (Fig. 7), and to show the internalization of H-Ft or transferrin in addition to just binding, multiple independent trials were not deemed necessary. |
| Randomization | For the in vitro inhibition (Fig. 1) and biolayer interferometry (Fig. 2) experiments, sample allocation was not randomized because the results were quantitative and did not require subjective judgment or interpretation. For in vivo efficacy studies, 3-week-old mice were sorted by sex (covariate) prior to infection so that all treatment groups consisted of equivalent percentages of males and females. For the progenitor study (Fig. 6), sample allocation was not randomized because BMMC are from the same donor for all conditions tested and the results were quantitative and did not require subjective judgment or interpretation. For the standard flow cytometry experiment (Fig. 7), sample randomization is not applicable because a single cell population of erythroblasts was used. Additionally, the results were quantitative and did not require subjective judgment or interpretation. For the imaging flow cytometry assay (Figs. 8 and 9), sample randomization is not applicable because a single cell line (MM.S1) was used. Additionally, the results were quantitative and did not require subjective judgment or interpretation. |
| Blinding | Investigators at Utah State University were not blinded for studies with the pathogenic JUNV Romero strain due to logistical reasons and limited personnel vaccinated with Candid#1 (requirement to work with the virus). Additionally, the results were quantitative and did not require subjective judgment or interpretation. The investigators at Harvard Medical School were not blinded during experiments or to outcome assessment because the results were quantitative and did not require subjective judgment or interpretation. Blinding is also not typically used in the field for similar biolayer interferometry-based competition assays. Researchers at UCLA were not blinded for the progenitor or flow cytometry studies. Counting of colonies for the progenitor assay was not blinded, but was performed by one person who is trained on the identification of such colonies. Blinding was not deemed necessary and is not typically used for flow cytometry studies as the |

results were quantitative and did not require subjective judgment or interpretation.

# Reporting for specific materials, systems and methods

We require information from authors about some types of materials, experimental systems and methods used in many studies. Here, indicate whether each material, system or method listed is relevant to your study. If you are not sure if a list item applies to your research, read the appropriate section before selecting a response.

## Materials & experimental systems

| n/a | Involved in the study |
|---|---|
| ☐ | ☒ Antibodies |
| ☐ | ☒ Eukaryotic cell lines |
| ☒ | ☐ Palaeontology and archaeology |
| ☐ | ☒ Animals and other organisms |
| ☒ | ☐ Human research participants |
| ☒ | ☐ Clinical data |
| ☒ | ☐ Dual use research of concern |

## Methods

| n/a | Involved in the study |
|---|---|
| ☒ | ☐ ChIP-seq |
| ☐ | ☒ Flow cytometry |
| ☒ | ☐ MRI-based neuroimaging |

## Antibodies

| | |
|---|---|
| Antibodies used | The antibodies targeting TfR1 (ch128.1/IgG1, ch128.1/IgG1 mutant and monoclonal 128.1), as well as the isotype negative control (anti-dansyl IgG1), were produced at University of California, Los Angeles (UCLA). See Materials and Methods for citations. The clone M-A712 mouse anti-human TfR1 antibody was purchased from, and validated by, BD Biosciences (Cat. No. 555534). |
| Validation | The antibodies targeting TfR1 (ch128.1/IgG1, ch128.1/IgG1 mutant and monoclonal 128.1), as well as the isotype negative control (anti-dansyl IgG1), were produced in murine hybridoma or myeloma cells grown in roller bottles and purified using affinity chromatography. They were validated using BCA assay to assess concentration and SDS-PAGE under non-reducing and reducing conditions to assess the molecular weight and proper assembly of light and heavy chains. In addition, binding to antigen and species specificity was assessed by ELISA and flow cytometry. See the Methods section of the paper for citations. |

## Eukaryotic cell lines

Policy information about cell lines

| | |
|---|---|
| Cell line source(s) | A549 human epithelial lung cells (ATCC CCL-185), Vero African green monkey kidney cells (ATCC CCL-81), HEK 293S GnTI-/- cells (ATCC CRL-3022), and MM.1S human myeloma cells (ATCC CRL-2974) were purchased from American Tissue Culture Collection (ATCC; Manassas, VA, USA). Human CD34+ cells used for differentiation into erythroblasts were purchased from HemaCare BioResearch Products. Human BMMC were purchased from STEMCELL Technologies. |
| Authentication | All cell lines obtained from the ATCC and are authenticated by short tandem-repeat (STR) profiling. |
| Mycoplasma contamination | All immortalized cell lines used tested negative for mycoplasma contamination. A549 and Vero cell lines used at Utah State University are tested quarterly with the PlasmoTest – Mycoplasma Detection Kit (InvivoGen, Cat. No. rep-pt1). The HEK 293S GnTI-/- used at Harvard Medical School are tested monthly for mycoplasma contamination using the e-Myco PCR Detection Kit (Bulldog Bio, Cat. No. 25234). The MM.1S cell line is routinely tested in the Penichet laboratory at UCLA using the MycoAlert Mycoplasma Detection Kit (Lonza, Cat. No. LT07-218). The human CD34+ cells from HemaCare BioResearch Products were tested by the company for a panel of infectious agents (including hepatitis B, hepatitis C, and HIV, but were not tested for mycoplasma. The CD34+ cultures were initiated upon receipt and lasted for ~6 weeks (including differentiation time). Human BMMC from STEMCELL Technologies were tested by the company for hepatitis B, hepatitis C, and HIV, but were not tested for mycoplasma. For the progenitor assay cells were thawed and the assays were immediately set up. The BMMC cultures lasted for 2 weeks. |
| Commonly misidentified lines<br>(See ICLAC register) | No commonly misidentified cell lines were used in the study. |

## Animals and other organisms

Policy information about studies involving animals; ARRIVE guidelines recommended for reporting animal research

| | |
|---|---|
| Laboratory animals | Mus musculus, human TfR1 knockin/mouse TfR1 knockout on a C57BL/6 and 129 hybrid background, male and female, 3-weeks of age. Mice were housed in a GM500 Green Line IVC system (Tecniplast SpA, Italy) in individually ventilated cages and fed hey were fed Harlan Lab Block and tap water ad libitum. Room air temperature in the biosafety level-3 enhanced laboratory dedicated to JUNV work was 72±4 °F with 30-70% air humidity. The room had a 12-12 h dark/light cycle. |
| Wild animals | The study did not involve wild animals |
| Field-collected samples | The study did not involve samples collected from the field |

| | |
|---|---|
| Ethics oversight | All animal procedures complied with USDA guidelines and were conducted at the AAALAC-accredited laboratory animal research facilities at Utah State University (USU) under protocol #10034, approved by the USU Institutional Animal Care and Use Committee. |

Note that full information on the approval of the study protocol must also be provided in the manuscript.

# Flow Cytometry

## Plots

Confirm that:

- [x] The axis labels state the marker and fluorochrome used (e.g. CD4-FITC).
- [x] The axis scales are clearly visible. Include numbers along axes only for bottom left plot of group (a 'group' is an analysis of identical markers).
- [x] All plots are contour plots with outliers or pseudocolor plots.
- [x] A numerical value for number of cells or percentage (with statistics) is provided.

## Methodology

| | |
|---|---|
| Sample preparation | Erythroblasts were differentiated from human bone marrow CD34 cells that were purchased frozen from HemaCare. No further processing of the CD34 cells was required. The CD34 cells were differentiated into erthroblasts as outlined in the Materials and Methods section of the manuscript. For the imaging flow cytometry studies, a human cell line (MM.1S) was used. The cells were purchased from ATCC and no further processing was needed. |
| Instrument | Standard flow cytometry: LSRII analytical flow cytometer. Imaging flow cytometry: Amnis ImageStream®X Mk II Imaging Flow Cytometer |
| Software | Standard flow cytometry data were aquired using FACSDiva™ (version 8.0.3; BD Biosciences) and histograms were created using FCS Express version 3.0 (De Novo Software). For imaging flow cytometry, the INSPIRE acquisition software (version 201.1.0.744; Luminex Corporation) and the IDEAS analysis software (version 6.2; Luminex Corporation) were used. |
| Cell population abundance | Does not apply. Cell sorting was not conducted. |
| Gating strategy | For standard flow cytometry the cell population was identified and gated using forward scatter (FSC) and side scatter (SSC) density plots. This live population gate was used to exclude debris (events with low forward and low side scatter). For imaging flow cytometry in focus events were gated by selecting events with a Gradient RMS value of greater than 50 on the brightfield channel. Single cell events were selected on the brightfield area versus aspect ratio density graphs, with single cells having an intermediate area value and a high aspect ratio value. Figures (Supplementary Figs. 2 and 3) exemplifying these strategies are provided in the Supplementary Information file. |

- [x] Tick this box to confirm that a figure exemplifying the gating strategy is provided in the Supplementary Information.
